# Healthcare provider perspectives on barriers and facilitators to integration of cardiovascular disease-related care into HIV care and treatment clinics in urban Tanzania

**DOI:** 10.3389/fpubh.2024.1483476

**Published:** 2024-12-24

**Authors:** Theresia A. Ottaru, Fileuka C. Ngakongwa, Zeeshan Butt, Claudia A. Hawkins, Sylvia F. Kaaya, Emmy O. Metta, Pilly Chillo, Helen N. Siril, Lisa R. Hirschhorn, Gideon P. Kwesigabo

**Affiliations:** ^1^Department of Epidemiology and Biostatistics, Muhimbili University of Health and Allied Sciences, Dar es Salaam, Tanzania; ^2^Department of Psychiatry and Mental Health, Muhimbili University of Health and Allied Sciences, Dar es Salaam, Tanzania; ^3^Phreesia, Inc., Wilmington, DE, United States; ^4^Department of Psychiatry and Behavioral Sciences, Feinberg School of Medicine, Northwestern University, Chicago, IL, United States; ^5^Robert J Havey Institute of Global Health, Feinberg School of Medicine, Northwestern University, Chicago, IL, United States; ^6^Department of Medicine, Feinberg School of Medicine, Northwestern University, Chicago, IL, United States; ^7^Department of Behavioral Sciences, Muhimbili University of Health and Allied Sciences, Dar es Salaam, Tanzania; ^8^Department of Internal Medicine, Muhimbili University of Health and Allied Sciences, Dar es Salaam, Tanzania; ^9^Department of Medical Social Sciences, Feinberg School of Medicine, Northwestern University, Chicago, IL, United States

**Keywords:** integrated care, cardiovascular diseases, HIV, CFIR, barriers, facilitators, ALHIV, Tanzania

## Abstract

**Background:**

The increase in the dual burden of HIV and cardiovascular diseases (CVD), calls for the provision of integrated HIV/CVD care. This study aimed to explore barriers and facilitators to the integration of HIV/CVD care within HIV care and treatment clinics (CTCs) in urban, Tanzania.

**Methods:**

Between March and April 2023, we conducted 12 key informant interviews with healthcare providers at six HIV CTCs in urban, Tanzania. Guided by the Consolidated Framework for Implementation Research (CFIR 1.0), we designed the interview guide and conducted a thematic analysis.

**Results:**

Out of the 11 CFIR constructs explored, three were barriers (cost, availability of resources, and access to information and knowledge), six were facilitators (complexity, relative advantage, patient needs, external policies and incentives, relative priority, and knowledge and belief about the intervention), and two (compatibility and self-efficacy) were both barriers and facilitators. Barriers to integration included a lack of equipment, such as BP machines, lack of space, unavailability of an electronic data-capturing tool at the HIV CTCs for monitoring CVD outcomes, and a shortage of trained healthcare workers, particularly in managing CVD comorbidities according to current recommendations. Providers acknowledged the increasing demand for CVD care among ALHIV and regarded integration as not a complex task. Providers reported that both services could be delivered simultaneously without disrupting client workflow and were determined to offer integrated care within HIV CTCs. Providers expressed concerns about medication costs and recommended that medications should be provided for free as part of the integrated care.

**Conclusion:**

Effective and sustainable HIV/CVD integrated care requires an understating of the existing barriers and facilitators within the HIV CTCs. This study identifies key barriers at HIV CTCs that must be addressed and facilitators to be leveraged before CVD care is integrated into HIV CTCs to ensure that CVD care is delivered effectively within an integrated system.

## Introduction

Cardiovascular diseases (CVD) remain the leading cause of morbidity and mortality worldwide (1). Adults living with HIV (ALHIV) are more likely to experience CVD compared to individuals without HIV infection (2). The excess CVD risk in this population is a result of a combination of behavioral risk factors (3, 4) and HIV infection-associated chronic inflammation and immune dysregulation (5). ALHIV are also reported to experience severe forms of CVD complications that manifest earlier compared to individuals without HIV infection (5, 6). As life expectancy increases among ALHIV, the burden of CVD among ALHIV is expected to rise significantly, threatening ALHIVs’ quality of life and overall survival (7, 8).

About 80% of the global CVD morbidity and mortality is reported in Sub-Saharan Africa (SSA) (9), a region that simultaneously has 70% of the global HIV burden (10). To address this double burden of disease, international health agencies and guidelines advocate for the integration of CVD prevention and care into HIV care and treatment clinics (CTCs), using the resources including providers and infrastructure of well-established HIV care programs (11, 12). Integration of CVD care into some HIV CTCs in SSA has been proven feasible (13, 14) and effective in reducing the overall CVD risk, through improving primarily blood pressure and glycemic control, and increasing patient satisfaction among ALHIV (15–17).

In Tanzania, about 1.7 million people are living with HIV (10). On the other hand, age-standardized total CVD death rate is estimated to be 13%, making it one of the highest in SSA. The percentage of disability-adjusted life years (DALYs) attributed to CVD is 5.1% for men and 4.6% for women (18). HIV infection is associated with a higher incidence of CVD, HIV prevalence among patients with stroke in Tanzania was 20.9% (19). Burden of CVD risk factors is also high among ALHIV in this setting; data suggest that 26.2–53.3% of ALHIV are hypertensive (BP ≥140/90 mm/Hg), 4.2–11.1% are diabetic (RBG > 11.1 mmol/L), 13.3% are obese and 20% have dyslipidemia (19–22).

The Tanzania national guideline for the management of HIV acknowledges the need to prevent CVD among ALHIV. Specific recommendations include patient education on lifestyle modification, screening, and management of the four most common CVD risk factors (hypertension, diabetes, dyslipidemia, and obesity) (23). Despite these recommendations, CVD care provided at the HIV CTCs in Tanzania remains inadequate (24). Our previous study on the experience of ALHIV with hypertension and/or diabetes while accessing care for these comorbidities in Tanzania (24) demonstrated that ALHIV do not receive routine hypertension and diabetes screening and management within HIV CTCs and mostly seek CVD care outside HIV CTCs. The need to attend multiple clinics causes loss of productive hours, increases transport costs, and contributes to treatment discontinuity. Furthermore, ALHIV with hypertension and diabetes do not receive adequate patient education or guidance on the management of these comorbidities (24).

The sustained and coordinated provision of CVD care is critical to ensuring the gains in life expectancy resulting from effective antiretroviral therapy (ART) are not reversed (25). Before CVD care is integrated into HIV CTCs, it is essential to understand barriers and facilitators that may influence the successful implementation and effective provision of integrated care. One study that reported on the barriers and facilitators to providing integrated HIV/CVD care in HIV CTCs in Tanzania, reported limited number of factors and did not use established frameworks (26). In this study, we applied the Consolidated Framework for Implementation Research (CFIR) (27) to formally explore providers’ perspectives on the barriers and facilitators to the integration of CVD care within Tanzanian HIV CTCs. CFIR is a robust and broadly used implementation science framework, designed to identify context-relevant barriers and facilitators for adapting interventions across multiple dimensions (27, 28). The use of the implementation science framework (CFIR) allows a formal methodology for selecting implementation strategies (29). Understanding the factors that influence integration is important for designing contextually appropriate HIV/CVD integrated care for ALHIV in Tanzania.

## Methods

### Study design

We conducted a qualitative study employing a phenomenological approach guided by CFIR to explore the perspectives of providers in HIV CTCs on the barriers and facilitators to the integration of CVD care into HIV CTCs in Dar es Salaam, Tanzania.

WHO defines integrated service delivery as *“The organization and management of health services so that people get the care they need, when they need it, in ways that are user-friendly, to achieve the desired results and provide value for money”* (30). In this study, we defined HIV/CVD integrated care as the provision of CVD care at the HIV CTC, which includes the provision of patient education on healthy lifestyle for CVD prevention, screening services, diagnosis, medication prescription (or referral services) for the three most common CVD risk factors: hypertension, diabetes, and obesity.

The phenomenology approach allowed in-depth exploration of the providers’ subjective experiences and perspectives (31, 32). In this study we adhered to the consolidated criteria for reporting qualitative research (COREQ): a 32-item checklist for interviews and focus groups (Supplementary file 1) (33).

### Study area and setting

This study was conducted in six high-volume HIV CTCs in Dar es Salaam Tanzania. These clinics were randomly selected from a list of 33 high-volume clinics (serving ≥1,500 clients) in the region and represent the three levels of Tanzania’s health care delivery system: Regional Referral Hospital (2), District Hospital (2), and Health Center (2). These HIV CTCs are part of our previous study that assessed cardiovascular health and experiences with care for CVD comorbidities among ALHIV (24, 34).

**Figure 1:**
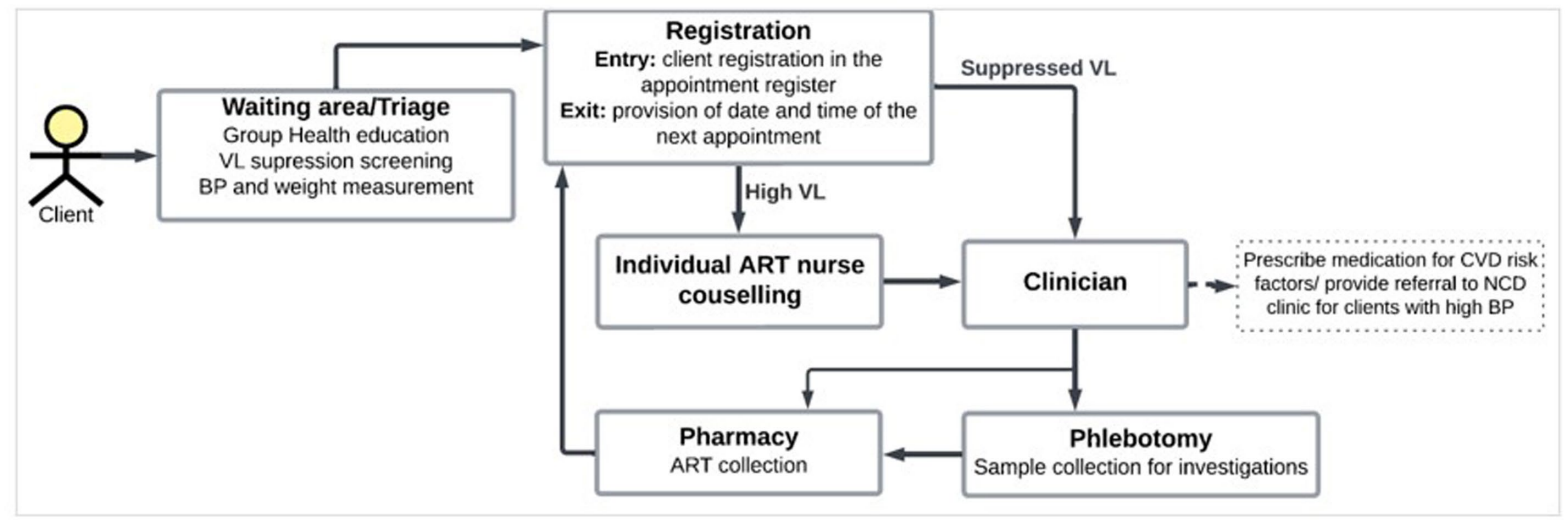
Workflow of patients at the HIV CTCs.

In Tanzania HIV CTCs, visits are scheduled between monthly and up to every 6 months, depending on how stable a client is on ART. Stable clients are defined as clients who are on ART for at least 6 months with no adverse reactions that require regular monitoring, no current illnesses (opportunistic infections (OIs) and uncontrolled comorbidities), have good adherence to ART and clinic visit appointments, and have no indication of immunological or virological failure (24). Figure 1 summarizes the workflow of clients at the HIV CTC. In addition to HIV-related care that includes ART dispensing, screening for opportunistic infections, HIV viral load monitoring, and ART adherence counseling, the guideline for the management of HIV recommends the provision of CVD care (24).

The guidelines recommend HIV CTCs clients receive patient education promoting a healthy lifestyle, undergo blood pressure and body weight measurements, and be screened for diabetes symptoms (polyuria, polydipsia, and polyphagia) at every visit. Blood glucose screening is required every 6 months while blood lipids screening is required annually (24). The guideline also recommends that providers prescribe medication for hypertension, diabetes, and dyslipidemia at the HIV CTC or refer clients with these CVD-risk factors to NCD clinics available at Regional Referral Hospitals and District Hospitals, and outpatient departments (Health Centers).

### Study participants

We purposively recruited 12 HIV CTC providers including one HIV CTC facility in-charge and one nurse supervisor from each of the six selected HIV CTCs. In addition to their administrative roles, all participants actively participated in providing care for clients at the HIV CTC during the study period. The proposed sample size was decided based on the recommendations from the literature (35), the available number of available providers with administrative roles within the HIV CTCs, and the researchers’ previous experience with qualitative studies involving providers within HIV CTCs.

### Data collection and procedures

We conducted 12 face-to-face key informant interviews (KII) between March and April 2023 using a semi-structured interview guide (Supplementary file 2) designed based on CFIR constructs (1.0) (27). This framework comprises five domains: intervention characteristics, outer setting, inner setting, characteristics of individuals, and implementation processes. We explored the four domains (11 constructs; Table 1), out of the five domains (39 constructs) of CFIR. We excluded the ‘implementation process’ domain because HIV/CVD integrated care has not yet been formally introduced at the HIV CTCs in Tanzania. The constructs explored were agreed upon through discussions with the study team. The selection was based on their relevance to the providers and the current state of HIV/CVD care integration at Tanzania HIV CTCs. The interview guides were piloted with three providers (one facility in charge and two nurse supervisors) at an HIV CTC within a district hospital that was not part of this study.

**Table 1:** CFIR framework domains and constructs used in the key informant interviews.

Domain	Construct	Definition
Intervention characteristic	Complexity	Perceived difficulty in the provision of CVD care alongside HIV care at the HIV CTC
	Relative advantage	Perceived benefit of integration of CVD care at the HIV CTC
	Cost	Perceived cost related to the provision of CVD care at the HIV CTC (cost of medication)
Outer setting	Patient needs	Extent to which ALHIV’s CVD needs are known and prioritized at the HIV CTCs
	External policies and incentives	Extent to which policies and regulations, external mandates, recommendations, and guidelines from the government or implementing partner support/advocate for the integration of CVD care at the HIV CTCs
Inner setting	Compatibility	Perceived alignment between the provision of integrated care and the care currently provided at the HIV CTC (workflow, systems, and processes)
	Relative priority	Perceived importance of integrating CVD care at the HIV CTCs
	Availability of resources	Extent to which resources are available/ allocated to facilitate the provision of CVD care at the HIV CTC including human resources, space, equipment, and electronic data management system
	Access to information and knowledge	Extent to which guidelines and training on the provision of CVD care are routinely available for providers at the HIV CTC
Characteristics of individuals	Knowledge and belief about the intervention	Providers’ attitude and values toward integration of CVD care at the HIV CTC including willingness and motivation to provide integrated care
	Self-efficacy	Providers’ perceived capability (competence, knowledge, and skills) to provide CVD care including screening, diagnosis, and management of CVD risk factors

CVD, cardiovascular diseases; CVD care, patient education, screening, diagnosis, and management of CVD risk factors.

In addition to the above, we also collected data on the participant’s age, highest education level, and years spent serving as the facility in-charge or nurse supervisor. TO (a female epidemiologist) and one research assistant (a male social scientist), both with experience conducting KIIs, conducted the interviews in Swahili language and took field notes. Interviews lasted 45–60 min and were audio recorded with the participants’ consent. The interviews were conducted in a private room within the HIV CTC where privacy and confidentiality were maintained. We monitored for emerging themes and by the eleventh interview, no new information was coming from the field (saturation). Audio recordings of the interviews were transcribed verbatim and translated into English language by the research assistant. TO and FN (a female co-author) reviewed the transcripts and the audio recordings to check for accuracy, consistency, and completeness.

### Data analysis

Data analysis began after the first two interviews and was part of an iterative process. First, TO and FN read the transcripts several times to familiarize themselves with the data. All transcripts were imported into Dedoose software for qualitative data management and analysis. TO and FN then coded the first three transcripts separately and developed the preliminary coding frame. Coding followed the thematic analysis technique (36–38). Deductive codes were drawn from the interview guide, which explored the selected CFIR constructs, while inductive codes were added as they emerged from the data. These codes were compared, and disagreements were resolved through a discussion with the study team until the final codebook was developed. TO coded the rest of the transcripts using the final agreed codebook, however, coding process was thoroughly reviewed by the research team, who are highly experienced in qualitative research. Repeating concepts reflecting the barriers and facilitators to integration of CVD care at HIV CTCs were coded and grouped into CFIR constructs (themes) and domains.

### Ethical considerations

The study was approved by the Muhimbili University of Health and Allied Sciences (MUHAS) MUHAS-REC-08-2020-343, National Institute for Medical Research (NIMR)-NIMR/HQ/R8a/VOL.IX/3513 and the Northwestern University (STU00214283) ethics committees. Data collected were de-identified and all participants provided written informed consent to participate including recording and anonymous quotation. Voluntary participation and confidentiality were maintained throughout the study. Participants were encouraged to ask questions at any stage of the research.

### Findings

#### Characteristics of the study participants

Out of the 12 participants, [median age of 46 years (range 38–58)], the majority were females (83%) and had served as facility in-charges or nurse supervisors for >5 years (50%). A summary of the characteristics of study participants is provided in Table 2.

**Table 2:** Characteristics of the study participants (*n* = 12).

Characteristic	Number (*n* = 12)
Age (median, range) (years)	46 (38–58)
	N (%)
Sex	
Female	10 (83)
Male	2 (17)
Facility type	
Health Center	4 (33)
District Hospital	4 (33)
Regional Referral Hospital	4 (33)
Cadre	
Medical Officer	4 (33)
Assistant Medical Officer	2 (17)
Enrolled nurse	3 (25)
Registered nurse	3 (25)
Level of education	
MD degree	4 (33)
BSc degree	3 (25)
Diploma	5 (42)
Years spent serving administrative role	
<1 year	2 (17)
1–5 years	4 (33)
>5 years	6 (50)

#### Overview of the key findings

The 11 constructs explored were classified as either barriers, facilitators, or both to the integration of CVD care at HIV CTCs. The CFIR constructs of cost, availability of resources, and access to information and knowledge were classified as barriers. The remaining constructs of complexity, relative advantage, patient needs, external policies and incentives, relative priority, and knowledge and belief about intervention were identified as facilitators, while compatibility and self-efficacy were identified as both barriers and facilitators (Supplementary file 3).

### Intervention characteristics

Three constructs under intervention characteristics were explored. These were *complexity, relative advantage, and cost*.

Regarding c*omplexity*, providers perceive that providing CVD care (patient education, screening, diagnosis, and management of CVD risk factors including hypertension, diabetes, obesity, and dyslipidemia) at the HIV CTC, is not difficult. This is because these providers already conduct some CVD risk factors screening such as BP and weight measurement, during each patient’s visit to the clinic. Some facilities have established an NCD integration room within the HIV CTC where a physician from the NCD clinic is available weekly to provide CVD services. In some facilities, BP levels are recorded in the database at the HIV CTC and are being monitored regularly.

*“I do not think it’s difficult because if we can measure a client’s blood pressure and body weight and then proceed with other services that (full integration) can also be done”* Participant 3, Regional Referral Hospital.

*“We have tried to allocate a room we term as an “integration room.” It is the room used by a clinician from the NCD clinic to see clients requiring integrated services (related to NCD) during clinic days. Therefore, I do not think it (providing HIV/CVD integrated care) is difficult”* Participant 1, District Hospital.

Related to *relative advantage*, providers reported that integrating CVD care at the HIV CTC would benefit clients in several ways. It would lead to early diagnosis and treatment of CVD risk factors, and allow holistic management and monitoring of patients.

*“If you tell clients that you are referring them elsewhere, they might go or not go. Sometimes they would say it is difficult to attend two different clinics. Therefore, there are times when they will not attend, others would tell you they have gone to a nearby hospital, hence monitoring becomes challenging”* Participant 2, District Hospital.

In terms of *cost*, providers reported that most patients face financial difficulties, are not covered by health insurance, and hence struggle to afford medication costs. Providers stated that without providing medications for free, integrated care may not yield significant benefits. Diagnosing and prescribing medications without providing them would not fulfill providers’ desire to help patients, particularly knowing that many cannot afford them.

“*Therefore, the main challenge is that most of our clients have low economic status, especially the clients with two or more diseases. So you tell the old man to go buy this medication outside and he says “alright my grandchild but I do not have the money.”* Participant 7, District Hospital.

### Outer setting

Two constructs under the outer setting were explored. These were *patient needs and external policies and incentives.*

Regarding patient needs, providers admitted that there is a high demand for CVD services among clients within HIV CTCs. Providers also added that CVD care needs are increasingly recognized and are gradually becoming a priority for clients at the HIV CTC.

*“There was a client with a very high blood pressure and it was clear that there was a need to have the clinic here. Another client asked the doctor; why they do not have the NCD services here (HIV CTC) so that they do not have to go to the other clinic after here (HIV CTC)”* Participant 2, District Hospital.

“*There was one with kidney issues, hypertension, and DM, attending a clinic at Muhimbili, so he was complaining that his clinics were scheduled on the same day here (HIV CTC) and at Muhimbili, so he came the day before for medications, he wished all services were provided at the same place.* Participant 4, Regional Referral Hospital.

*“For every adult client, we measure their blood pressure. We did not do this in the past, but since we began this quarter in October we have set up a triage. Before a client receives any service, we must measure their blood pressure.* Participant 1, District Hospital.

While providers report that CVD care, especially screening for hypertension, is increasingly being prioritized, they admitted that HIV care is still the main priority at the HIV CTC. One provider stated that this is because, with the current infrastructure, they are not equipped to monitor CVD risk factors as they do with HIV. Additionally, medications for hypertension are not provided at the HIV CTCs and because they are expensive, providers are never certain whether the patients have procured them after they were prescribed.

*“Yes, they (hypertension and diabetes) are prioritized but we cannot monitor them the way we monitor ARTs. First, you are not sure if the patient has taken the medications; at most, you have not even dispensed the drug to the patient. You cannot ask the patient why he has not taken medications, (he does not have money)”* Patient 3, Regional Referral Hospital.

With regards to *external policies* providers reported that it is currently mandatory for every patient visiting the HIV CTC to undergo BP and weight measurement, which must be recorded in the HIV CTC database (CTC2 database). Efforts to implement these recommendations have accelerated with implementing partner support (Management Development for Health – MDH). MDH distributes BP machines, monitors the completeness of BP data in the database, and provides feedback to the HIV CTCs.

“*Yes, they (MDH) have given us 4 blood pressure machines and 2 weighing scales, the big monitor you see there, also batteries sometimes are provided by the hospitals but sometimes there is a delay from procurement, so they also give us batteries”* Participant 4, Regional Referral Hospital.

“*Blood pressure and even the blood glucose columns have been added to the new CTC2 card, so we cannot leave the column blank. In the past, there was no follow-up for hypertension and diabetes but now we follow up.* Participant 9, Regional Referral Hospital.

Providers added that the introduction of multi-month dispensing (MMD) of ART (at 3 and 6 months MMD) has significantly reduced the daily patient load at the HIV CTC. This provides an opportunity to introduce CVD care services as an additional service at the HIV CTC.

*“We give a fixed number of pills let us say 90 so there are some days where the number of clients is very high, but there are days we have few patients like 20 or forty. Currently, the patients are not as many as they used to be in the past”* Participant 12, Regional Referral Hospital.

Another provider warned that patients on the MMD scheme might need to visit the clinic monthly for CVD care, which could cause the clinic to become overcrowded with clients as it was in the past.

*“A stable client comes for his medications after six months and if you ask him to come monthly for hypertension monitoring, we will have congested clinics as before”* Participant 4, Regional Referral Hospital.

### Inner setting

Four constructs under the inner setting were explored. These were *compatibility, relative priority, availability of resources, and access to knowledge and information.*

In terms of *compatibility,* providers reported that the current setup of services at the HIV CTC (Figure 1) allows for the seamless integration of CVD services without disrupting patient workflow. Integration will not be a problem if both services are provided concurrently.

*“I honestly do not see any problem, unless it was a service that you provide at a separate time. But if I see them at once, they can come with their test results, and I can review the clinical notes to see what s/he received from the beginning. I do not think that will be a problem”* Participant 7, District Hospital.

However, providers added that the electronic data-capturing tool at the HIV CTC (CTC2 database), accommodates only a few non-HIV outcomes such as BP measurements. Data on other CVD risks such as glucose and medications for these conditions are not required to be recorded. The main hospital database which captures all outcomes including medication prescribed, is not installed at the HIV CTC, and the HIV CTC and the main hospital database are not linked. Providers emphasized that linking the two databases would ensure that they can attend to patients with CVD comorbidities at the HIV CTC and record their progress in the main hospital database for more efficient monitoring and documentation.

“*At our center apart from cost, the main issue is the integration of the systems, meaning that when a client comes in, he should first be registered in the hospital registration system so that he is not required to register again when he needs to be seen by a doctor for CVD services, that will help in everything, all patient could receive services here (HIV CTC)”* Participant 9, Regional Referral Hospital.

Regarding *relative priority,* providers stated that the introduction of CVD services at the HIV CTC is considered important as it will allow the simultaneous provision of highly needed CVD care alongside HIV care. Integration will consequently reduce the time and money patients spend traveling between clinics.

*“It will save them time, instead of coming here and going elsewhere. If the client comes here to the clinic, the client knows that s/he will receive all services at once. So, it will minimize cumbersome movements”* Participant 1, District Hospital.

Related to the *availability of resources,* providers at lower-level health facilities (District Hospitals and Health Centers) reported a lack of space and human resources to provide CVD care alongside HIV care. However, this was not the case for providers at Regional Referral Hospitals.

*“There is not enough space because the building is small even if you say we should have more staff the rooms will not be enough. But the staff are not enough as well but we are doing our best”* Participant 10, Health Center.

Providers added that integration might increase their workload, which could subsequently compromise the quality of care currently provided at the HIV CTC and extend patient waiting times.

*“It will not be difficult in such but there will be more work for all of us including the pharmacist, he must spend more time on dispensing other medication”* Participant 3, Regional Referral Hospital.

*“Difficulty could be with buying medications and staff because we are not only dealing with HIV, clients with NCDs will also need to see a doctor so it can be exhausting and also jeopardize efficiency.”* Participant 4, Regional Referral Hospital.

Providers also emphasized that successful integration requires ensuring the availability of all necessary equipment (BP machines, glucometers, and RBG strips), diagnostic tests (blood chemistry), and medications. Such resources are currently lacking at the HIV CTCs.

*“We have no equipment like RBG machines, but it would be good if we had the machine, if we find a symptomatic client we link them with the NCD clinic at OPD”* Participant 1, District Hospital.

Regarding *access to information and knowledge,* half of the providers reported receiving training on CVD comorbidities management, mostly from regional referral and District Hospitals. The remaining half reported not being trained in NCD management, being unaware of the current recommendations, and not having guidelines for managing these conditions. These providers rely on physicians from the NCD clinics for guidance on managing CVD comorbidities and refer patients to them for treatment.

*“Okay, apart from the knowledge I got from school long ago, I received this ‘crash training,” maybe we remind each other in meetings on the first or second-line management of hypertension. But if I am worried, I will consult the physician from there (NCD clinic) since he is more experienced, I describe the client’s case and I get advised on how to proceed”* Participant 1, District Hospital.

### Characteristics of individuals

Two constructs under the characteristics of individuals were explored. These were *knowledge and belief about intervention and self-efficacy.*

Related to *knowledge and belief about the intervention*, providers expressed their willingness and motivation to provide CVD care alongside HIV care, despite a potential increase in workload, and the existing challenges of staff shortages and limited space at the HIV CTC.

*“Yes, it’s something that I wish for to happen because it’s good to know the outcome when you are taking care of a patient, but if you only give ART and this patient is hypertensive and diabetic, he will take the ART, but what about the other aspects that you were not able to help him with?”* Participant 3, Regional Referral Hospital.

Regarding *self-efficacy*, providers reported that their long-standing focus has primarily been on HIV care, ART adherence, and viral suppression. Although they are confident in their ability to screen for CVD comorbidities, they reported the need for training, particularly on current management recommendations for these comorbidities.

*“I would request refresher training, regarding the new guidelines. They should send new employees for training or do on job training. Guidelines will also assist in making diagnoses and choices of treatment. There should be regular refresher training and mentorship which will help a lot”* Participant 6, Health Center.

## Discussion

We conducted a qualitative study with 12 HIV CTC providers at three diverse healthcare facility levels to understand the barriers and facilitators to the integration of CVD care within the usual HIV care in urban Tanzania. The major barriers to integrating CVD care in HIV CTCs identified in this study were related to the inner setting domain. Providers reported a lack of resources to provide CVD care alongside HIV care within HIV CTCs. These resources included (functioning) equipment for screening CVD comorbidities (BP machines and glucometers), trained human resources on CVD comorbidities management, and electronic data capturing tool for monitoring CVD comorbidities management and outcomes. Qualitative studies of integrated HIV and CVD care in SSA reported similar challenges (26, 28, 39, 40).

One additional barrier that was reported among providers in our study that was not reported in previous studies is the fear of increased workload after HIV/CVD care integration. Providers in this study expressed their concerns about HIV CTCs becoming overwhelmed again, as they were before the introduction of MMD. Integrating CVD care into HIV CTCs may potentially overburden providers and lead to congestion at these clinics (41). Ministry of Health needs to obtain effective buy-in from providers before integration, as the integration of HIV and CVD care might increase their workload. However, clients with controlled CVD risk factors such as hypertension and diabetes can also be seen for up-to 6 months if their comorbidities are well controlled, and the supply chain allows for multi-month prescribing for CVD risk factors medications. With good medication adherence and self-management strategies, clients will eventually transition to MMD for CVD risk factors management, just as they did for HIV. In previous research, HIV CTC providers were reported to be less willing to provide CVD care, citing that CVD care is not the traditional role of an HIV CTC provider (42). It is essential to engage the HIV CTC providers in the planning process for HIV/CVD integration to ensure that all concerns are addressed before moving forward with the integration.

Providers from lower-level health facilities, particularly Health Centers, reported a lack of space to provide both CVD and HIV care simultaneously. This was not the case for Regional Referral Hospitals. This suggests that higher-level health facilities may be better suited for initial small-scale implementation science trials to assess implementation and clinical outcomes of HIV/CVD integrated care, informing the design of an effective and sustainable model in Tanzania. Future research can focus on developing implementation strategies based on our study findings and evaluating a context-appropriate HIV/CVD integrated care model.

Although HIV care in Tanzania, including doctor consultations and medication, is provided for free, care for CVD comorbidities is not. Providers stated that without providing CVD care for free for ALHIV including medications, integrated care may not yield significant benefits. Provision of free medication may motivate clients to remain in CVD care, however, the long-term sustainability of this approach is a concern. Research has also shown provision of free CVD comorbidities medication as a component of HIV/CVD integrated care, may not necessarily improve clinical outcomes (43). Our previous study exploring experiences with hypertension and diabetes management found that there is intermittent medication use for these conditions among ALHIV. Beyond medication cost, this was due to negative perception regarding CVD risk factor medications. They reported fears of side-effects associated with the life-long use of hypertension and diabetes medication alongside HIV treatment, and the perceptions that medications for these conditions are ineffective (24). Successful integration with positive clinical outcomes requires efforts beyond providing free medication, including regular monitoring of patients, medication adherence, and lifestyle counseling (44). The exploration of financing options for integrated HIV/CVD care among HIV CTC providers was beyond the scope of this study. Our future plan is to engage policy makers at the Ministry of Health and implementing partners to understand existing plans for financing CVD care at the HIV CTCs. This understanding is critical for informing design and testing of the integrated HIV/CVD care.

Despite the reported barriers, our study identified a number of facilitators that could support a successful HIV/CVD care integration in Tanzania. HIV CTCs in our study reported that the implementing partner (MDH) has started to support CVD care within their HIV CTCs. MDH provides BP machines and monitors, trains healthcare workers on NCD management, and ensured that BP is among the indicators recorded and monitored at the HIV CTCs. With this support, there is now more screening for hypertension in these HIV CTCs compared to the past, and those diagnosed are being monitored. This facilitating factor is among the factors that was not reported by the previous study in this setting (26). These initiatives establish a strong foundation for integration. Scaling up to include other CVD comorbidities, such as diabetes, and replicating these efforts among other implementing partners across Tanzania is essential.

HIV CTC providers in our study maintained a positive attitude toward integrating CVD care into the HIV CTCs and did not consider it a complex task. Providers acknowledged the growing need for CVD comorbidities care among their clients and reported being motivated to meet this need. A previous study in Tanzania also reported the presence of a health workforce willing to provide CVD care at the HIV CTCs (26). The availability of a motivated healthcare workforce ready to provide integrated care offers a valuable opportunity for the Ministry of Health, and other stakeholders in Tanzania, to establish the necessary environment for integration. There is a growing body of evidence that integrating CVD care within HIV CTCs is feasible in SSA (13, 14). The guideline for the management of HIV in Tanzania also recommends the provision of CVD care at HIV CTCs (24). However, integrating care without adequate resources may compromise the quality of the existing HIV care (45).

### Strengths and limitations

This study systematically explores barriers and facilitators to integrating CVD care within HIV CTCs using an implementation science framework (CFIR). This approach enhances cross-study comparisons and enables systematic mapping of implementation strategies and designing of an integrated model using implementation science research approaches (46). We involved three different healthcare levels (Regional Referral Hospitals, District Hospitals, and Health Centers) and identified variations in barriers and facilitators by these levels. However, the study had a few limitations. Firstly, the six HIV CTCs recruited in this study are supported by the same implementing partner and are in urban settings. As a result, our findings may have limited generalization to HIV CTCs supported by other implementing partners who might be providing different types or levels of support to their HIV CTCs. Future research could purposively select HIV CTCs supported by different implementing partners in Tanzania to explore variations in the level of support provided for CVD care. Findings presented in this paper include only providers’ perceptions of the factors that could influence the integration of CVD care at HIV clinics and lack representation of patients’ voices. However, the experiences, perceptions and preferences of ALHIV regarding HIV/CVD care integration that could potentially influence integration have been described in our previous publication (24).

## Conclusion

In parallel with ongoing efforts to implement the provision of CVD care within HIV CTCs in Tanzania, this study highlights key barriers including lack of equipment, lack of space, unavailability of an electronic data-capturing tool for monitoring CVD outcomes, and a shortage of trained healthcare workers. The barriers should be considered when designing a context-relevant HIV/CVD integrated care model for ALHIV, leveraging existing resources and facilitators within the HIV CTCs.

## Data Availability

The raw data supporting the conclusions of this article will be made available by the authors, without undue reservation.

## Supplementary material

Supplementary FiLe 1 COREQ checklist.

Supplementary FiLe 2 Interview guide.

Supplementary FiLE 3 Barriers and facilitators to integration of CVD care at HIV CTCs in Dar es Salaam, Tanzania 2022.
