# Supplementary material for: Healthcare provider perspectives on barriers and facilitators to integration of cardiovascular disease-related care into HIV care and treatment clinics in urban Tanzania
Source: Front Public Health. 2024 Dec 24;12:1483476. doi: 10.3389/fpubh.2024.1483476 (PMC11703862; doi:10.3389/fpubh.2024.1483476)
Supplement: Supplementary FiLe 2 — Interview guide. [file Table_2.DOCX]

Qualitative interview guide for health care providers in English

Thank you for agreeing to participate in this interview. As I previously mentioned, the purpose of this study is to understand the barriers and opportunities for integration of cardiovascular related services (specifically for hypertension, diabetes, dyslipidemia and overweight/obesity) at the care and treatment clinics (CTC) for HIV in Tanzania. You are being asked to participate in this study because you are a health care worker providing care for people living with HIV at the CTC. Information obtained from this study will be used to improve cardiovascular disease care for people living with HIV.

As the reminder this interview will be audio recorded, and all the recordings and information obtained will remain confidential and without any identifying information.

Do you have any additional question before we start?

**Introductory questions**

1. What is your job title/role?
2. What are your main responsibilities at the CTC?
3. For how long have you been working at the CTC?

**Current situation**

I would like to start by having you briefly describe the care for CVD risk factors and/or diseases delivered at your facility (CTC).

1. What can you tell us about CVD care that is currently provided at your facility?

- Briefly describe how you are involved in the care and management of persons with CVD risk factors and/or diseases at your clinic (responsibilities and involvement)
- Prompt about availability of services (screening, diagnosis, treatment or referrals and health education) for CVD risk factor and/or diseases

**Strengths and weakness of CVD care integration at HIV clinics**

1. In your view, what are the **current strengths** of the care delivered for CVD risk factors and/or diseases for ALHIV attending your CTC?

- Do you think the delivery of CVD related care is/will be effective in your clinic? Why or why not?
- What is working well at the moment?

1. In your view, what are the **main weaknesses** of the care delivered for CVD risk factors and/or diseases for ALHIV attending your HIV clinics?

- What is not working well at the moment?

1. Please, describe the referral system for patients with CVD risk factors and/or diseases.
2. What type of referral system is in place at your facility for patients with CVD risk factors and/or diseases?
3. What are the strengths and weaknesses of this system?

**Complexity**

1. What are the challenges with the process of delivering care for CVD risk factors and/or diseases at your clinic?
2. Please consider the following aspects of the intervention: duration, scope and number of steps involved
3. To what extent are the resources adequate for delivering this type of service?
4. Are there enough trained staff, diagnostic tools, medication, and space at the HIV clinic?
5. Do you follow any guidelines for the care of persons with CVD risk factors and/or diseases? (are the guidelines available?)
6. Have you attended any trainings at your facility or outside on care for CVD risk factors and/or diseases among ALHIV? If so, please describe them.

**Self-efficacy**

1. How confident are you that you will be/are able to deliver care for persons with CVD risk factors and/or diseases for your clients at the clinic?
2. What gives you that level of confidence (or lack of confidence)?
3. What makes it easier for you? What is harder?

- What about your colleagues, how confident do you think your colleagues feel about delivering care for CVD risk factors and/or diseases for clients at your clinic?

**Beliefs about the intervention**

1. How do you feel (perception) about delivering care for CVD risk factors and/or diseases at your clinic?
2. Do you look forward to providing such services? Are you eager to do so? Stress? Enthusiasm? Why do you fell so?
3. What about other providers? What is the general level of receptivity among other providers at your clinic?

**Need for integration of care for CVD risk factors and/or diseases in the clinic**

1. Do your clients express the need for CVD risk factors and/or disease care? What is the perceived need for CVD risk factor and/or disease care in your HIV clinic

- How will the provision of these services help these patients? E.g., improved access to services? Reduced waiting time? Help with self-management? Reduced travel time and expense?
- In your opinion, how will the clients respond to these services?

**Relative advantage** *(important if those services are not at the moment provided at the clinic)*

1. What advantages does the delivery of CVD related care have compared to existing programs? Are there disadvantages?
2. What challenges will the clients face while accessing such services?

**Suggestions for improvement**

1. What would make the process of delivering care for CVD risk factors and/or diseases better?
