# Supplementary material for: Healthcare provider perspectives on barriers and facilitators to integration of cardiovascular disease-related care into HIV care and treatment clinics in urban Tanzania
Source: Front Public Health. 2024 Dec 24;12:1483476. doi: 10.3389/fpubh.2024.1483476 (PMC11703862; doi:10.3389/fpubh.2024.1483476)
Supplement: Supplementary FiLE 3 — Barriers and facilitators to integration of CVD care at HIV CTCs in Dar es Salaam, Tanzania 2022. [file Table_3.docx]

**Supplementary file 3:** Identified barriers and facilitators to integration of CVD care at HIV CTCs in Dar es Salaam, Tanzania 2022

| CFIR domain and constructs (themes) | Barrier or facilitator | Explanation | Representative quotation |
| --- | --- | --- | --- |
| **Intervention characteristics** | | | |
| Complexity | Facilitator | Provision of integrated HIV/CVD care is not perceived as difficult, as some of the CVD services are already provided at the HIV CTC | “*We provide some of these services (CVD care) because measuring blood pressure, weight, and height is mandatory. In case it (blood pressure) is elevated than normal, we can prescribe medication or refer the client to the NCD clinic since it is within our facility”* Participant 7, District Hospital |
| Relative advantage | Facilitator | Provision of CVD care at the HIV CTCs would enhance the holistic management and monitoring of patients and ensure care continuity | *“For example, you have diagnosed a patient with hypertension and you cannot follow up the patient, it feels like you have just diagnosed the patient and dumped him. But if you can make follow-up and you are able to assess whether to give medications weekly or after two weeks, or if stabilized monthly, it makes sense. Like how we follow up with patients here on their CD4 counts and viral load. If we would just be checking CD4 and viral load without any intervention, it would not make sense and we would be managing halfway”* Participant 2, District Hospital |
| Cost | Barrier | Cost of care including the cost of medication poses a potential barrier if CVD services are not provided for free at the HIV CTC | “*Something that makes me worried is when the client will not be able to access the service because they are unable to pay for it. Also, I will not be at peace if we are just diagnosing the condition and can’t further help because the patient can’t pay, we rather not offer the services at all. It gives you tension as a health care provider”* Participant 3, Regional Referral Hospital |
| **Outer setting** | | | |
| Patient needs | Facilitator | There is a high demand for CVD care among ALHIV | *“We are lagging behind a bit with these NCDs because there are clients who come for their medications (ARTs) and with blood pressures of 200/120 and they are to be seen next after 6 months without any intervention, likely, this patient won’t be on the same health condition, in a month or two he might have a stroke. We have a lot of clients with hypertension and diabetes.”* Participant 9, Regional Referral Hospital |
|  |  | Providers are aware of the need for CVD care among clients, there is increasing prioritization of CVD services at the HIV CTC | *“There is also increased awareness compared to the past when we never talked about the NCDs but currently we do, even when a patient goes to see the doctor without the blood pressure reading he will ask the patient to go back and get his blood pressure checked”* Participant 2, District Hospital |
| External policies and incentives | Facilitator | Support from Implementing Partner to equip clinics with BP machines and scales, and ensure regular monitoring of BP and weight at the HIV CTC | *“They (MDH - Management Development for Health) support us by providing machines in case we don’t have them. For example, sometimes we see about one hundred patients and we have a single machine which can sometimes display an ‘error’ reading. So they provided a monitor since it can measure many clients. They also follow up closely to ensure that clients are having their blood pressure, weight, and height measured and recorded in the CTC2 database”* Participant 2, District Hospital |
| **Inner setting** | | | |
| Compatibility | Facilitator | Workflow of patients at the HIV CTC allows concurrent provision of both CVD and HIV care | *“I honestly don’t see any problem, unless it was a service that you provide at a separate time. But if I see them at once, they can come with their test results, and I can review the clinical notes to see what s/he received from the beginning. I don’t think that will be a problem”* Participant 7, District Hospital |
|  | Barrier | The HIV CTC database and the main hospital database operate independently | *“The challenge comes because there is no link between the CTC2 database (HIV CTC database) and the hospital database, so we send data manually to the hospital system”* Participant 2, District Hospital |
| Relative priority | Facilitator | HIV/CVD care integration enables the simultaneous provision of both services and reduces travel | *“Firstly, it will minimize client movement. That is, when they come to the clinic (HIV CTC) they will receive all services. The same clinician attending to the client addresses all issues at once. We can have files that identify clients with DM or hypertension so that the doctor or counselor can know that this client requires both ARTs and hypertension or diabetes medication, such that the client receives all services at once.* Participant 1, District Hospital |
| Availability of resources | Barrier | Limited resources such as equipment, space, and healthcare workers especially at District Hospitals and Health Centers | *“We only have one BP machine and sometimes all of a sudden, the machine signals battery low. Then you have to start the process by tracing the procurement officer and all, therefore, there will be some clients who will be attended to without measuring their blood pressure since there is only one machine”* Participant 7, District Hospital |
| Access to information and knowledge | Barrier | Lack of training and guidelines on CVD risk factors management, among providers in the District Hospitals and Health Centers | *“Although we are also trained on non-communicable diseases, we are not yet trained on medications. So, we check blood pressure, and educate them. When a client has high blood pressure, we refer him/her to the OPD”* Participant 5, District Hospital  *“To be honest there are no specific NCD or hypertension guidelines, let us say it is based on the knowledge I gained from school”* Participant 2, District Hospital |
| **Characteristics of individuals** | | | |
| Knowledge and belief about the intervention | Facilitator | Providers are eager and motivated to provide CVD care alongside HIV care | *“But here we will monitor everything, although there will be more work, the patient will benefit from it and the aim is to save lives.”* Participant 3, Regional Referral Hospital  *“I really do wish it (CVD care) comes, if it is all integrated under the umbrella of HIV care and clients receive free medication, I believe we will help a lot of clients”* Participant 1, District Hospital |
| Self-efficacy | Facilitator | Providers have a strong belief in their ability to monitor CVD comorbidities | “*Currently, to a greater extent (of screening for hypertension), can be above eighty-five percent, unlike previously where there were healthcare providers who did not give it much attention. I am a self-appointed focal NCD person, I follow it up. A client must be measured upon arrival and documented in a registration book. We crosscheck if the providers document or if there is any challenge”.* Participant 2, District Hospital |
|  | Barrier | Need for more training on the management of CVD comorbidities especially among providers at lower-level facilities | *“I would request refresher training, regarding the new guidelines. They should send new employees for training or do on job training. Guidelines will also assist in making diagnoses and choices of treatment. There should be regular refresher training and mentorship which will help a lot”* Participant 6, Health Center |
